# How does the continued use of the mask affect the craniofacial region? A cross‐sectional study

**DOI:** 10.1002/brb3.3077

**Published:** 2023-06-26

**Authors:** Elena Marques‐Sule, Gemma Victoria Espí‐López, Lucas Monzani, Luis Suso‐Martí, Miriam Calderón Rel, Anna Arnal‐Gómez

**Affiliations:** ^1^ Department of Physiotherapy, Faculty of Physiotherapy University of Valencia Valencia Spain; ^2^ Physiotherapy in Motion, Multispeciality Research Group (PTinMOTION), Department of Physiotherapy University of Valencia Valencia Spain; ^3^ Exercise Intervention for Health (EXINH) University of Valencia Valencia Spain; ^4^ Ivey Business School at Western University London Ontario Canada

**Keywords:** face mask, headache, impact, pandemic, protection, quality of life, SARS‐CoV‐2, temporomandibular joint

## Abstract

**Objective:**

The aim was to compare the effects between pre‐pandemic mask‐free living versus pandemic‐related continuous mask use.

**Methods:**

A retrospective study was carried out. This study was conducted with 542 face mask users. Assessments included presence, frequency and impact of headache, temporomandibular disorders, and quality of life (QoL).

**Results:**

Continuous mask use had a large main effect on headache, temporomandibular pain, and QoL (*p* < .0001; *d* = 1.25), but this effect was nuanced by mask type. Participants who declared suffering from headache increased by 84% with cloth masks, and by 25% with FFP2 masks. Temporomandibular pain increased by 50% and by 39% when wearing surgical masks and FFP2, respectively (*p* < .06; *d* = .19). The mask type did not nuance the effect on headache impact (*p* > .05; *d* = .06). QoL decreased regardless of mask type (*p* < .05; *d* = .21), the decrease being 38% for surgical masks, and 31% for either cloth or FFP2 masks.

**Conclusions:**

Continuous mask use, regardless of type, increased existence of headache, headache impact, temporomandibular pain, and reduced QoL.

## Graphical abstract

‐The continuous use of the mask negatively affects the temporomandibular joint, the headache and its impact. ‐ The type of mask influences these variables in different ways, cloth masks affect more, followed by surgical masks and FFP2. ‐ The continued use of the mask affects quality of life, regardless of the type of mask.

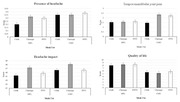

## 1 INTRODUCTION

Coronaviruses are a family of viruses that can cause diseases such as common cold or severe acute respiratory syndrome (SARS). In 2019, a new coronavirus was identified as the cause of an outbreak of diseases that originated in China. This virus is currently known as SARS coronavirus 2 and the disease it causes is the coronavirus disease 2019 (COVID‐19). In March 2020, the World Health Organization declared the outbreak of this disease as a pandemic whose consequences are still suffered (World Health Organization, 2019).

Therefore, the health care area has been going through difficult times. As a result, it has been necessary to implement a series of prevention standards in relation to health safety, such as personal protective equipment. Among the multiple protection measures, health authorities and governments established the mandatory continuous use of facemasks (CMU) as a massive protection measure against the virus, making necessary its compulsory use (Greenhalgh et al., 2020; Howard et al., 2020; World Health Organization, 2019).

During COVID‐19 pandemic, and after that period, health care professionals have continued using facemasks. Although this protection against the disease is necessary, CMU could have secondary repercussions in different health areas. In this regard, several studies have been carried out in health care professionals relating the use of facemasks and headaches. The most used facemasks are filtering face pieces (FPP2, N95, and KN95), as well as surgical facemasks. The relationship of facemask use with altered, dizziness, and headache has been demonstrated (Kyung et al., 2020). In fact, the “de novo” primary headache has been related to the impact of facemask use in the working, family, personal, and social spheres (Ramirez‐Moreno et al., 2021), as well as with sleepiness, headaches, and psychological symptoms (Cigiloglu et al., 2021). However, other authors have observed that in health care professionals with preexisting headache, the pain increased when using facemask and was also related to symptoms, such as tachypnea, sleep disorders, and fatigue (Köseoğlu Toksoy et al., 2021). Nevertheless, only health care professionals were included in these studies, thus no extrapolation to general population could be done. In addition, the relationship with quality of life (QoL) or with headache impact was not studied.

CMU can have an impact on the musculoskeletal system (Lim et al., 2006) and, specifically wearing this protective element may have myofascial effects on the craniofacial muscles. In this sense, CMU, as well as other health protective equipment, could create tensions and provoke or increase headache (Marfil Rivera et al., 2021), and as a consequence, produce a pain‐related impact. On the other hand, previous studies have shown that a slight variation in the position of the temporomandibular joint (TMJ) is related to the onset of headache (Di Paolo et al., 2017; Reik & Hale, 1981). All this could have a global impact on peoples’ QoL. To the best of our knowledge, there are no studies that assess the association of CMU with symptoms related to the TMJ.

Therefore, it is necessary to accurately establish consequences in the craniofacial sphere arising from CMU, not only in health professionals who wear CMU in their daily practice but in the general population, and to be able to act accordingly and with greater knowledge from the physiotherapy perspective and with more appropriate treatments.

It was hypothesized that COVID‐19 prevention CMU has an impact on people in different areas. First, mask use influences variables such as headache, TMJ discomfort and pain, headache impact, and QoL and, in turn, may be contingent on the frequency of headaches. On the other hand, the use of different mask types can influence these variables to a greater or lesser extent. The main objective of the present study was to compare the differences of CMU against mask‐free living (MFL) on headache, TMJ discomfort and pain, headache impact and QoL, and to analyze the impact of different mask types.

## 2 METHODS

### 2.1 Participants

Volunteers who participated in this study were over 18‐year old and were wearing facemasks daily for the avoidance of COVID‐19 during at least 2 h/day and had the ability to answer the questions and complete the questionnaires. Subjects with musculoskeletal injuries in the craniofacial region, institutionalized subjects, as well as subjects who wore masks for less than 2 h a day were excluded from the study.

### 2.2 Study design

A retrospective study was carried out during the months of February to March 2021 taking into account mandatory CMU due to the current pandemic. The study was conducted at the (*blinded*) and consisted of an *online* questionnaire which included sociodemographic characteristics, ad hoc questions, and specific evaluation instruments for each variable. The study included an interview regarding aspects related to headache and its impact, TMJ pain and QoL. It was conducted according to Declaration of Helsinki and was recorded in ClinicalTrials.gov Identifier: NCT04727762.

### 2.3 Ethical considerations

The study was approved by the Human Research Ethics Committee of the (blinded) (UV‐INV_ETICA‐1517574) and followed the ethical principles according to the Declaration of Helsinki. Participants signed an informed consent guaranteeing confidentiality prior de starting the study.

### 2.4 Outcome measures

Sociodemographic characteristics were collected (age, gender, marital status, education, and occupation), as well as face mask type which participants used. In addition, the assessment included headache characteristics, TMJ discomfort and pain, impact of headache and QoL. All outcomes were assessed comparing current CMU against pre‐pandemic MFL.

#### 2.4.1 Primary outcomes

##### 2.4.1.1 Headache

An ad hoc was conducted which included a closed‐ended question about the existence of headache in CMU and MFL situations with two possible answers (yes, no). If participants responded affirmatively, the following headache characteristics were assessed: (a) frequency (once a month, once a week, once a day); (b) onset (gradual, sudden); (c) description (pressing/tightening quality, pulsating quality); (d) intensity (low, moderate, intense); (e) duration (minutes, hours, days); (f) remission (progressively, abruptly, not remission); (g) location (occipital, frontal, orbicular, vertex); (h) time of onset (afternoon/morning, evening, night).

##### 2.4.1.2 Headache impact

Measured by the Headache Impact Test‐6 (HIT‐6) (Kosinski et al., 2003; Ware, 1999), the HIT‐6 was designed to provide a global measure of the adverse impact of headache. This questionnaire measures the adverse impact of headache on social functioning, role performance, vitality, cognitive functioning and psychological distress, and scores as follows: never (6 points), hardly ever (8 points), sometimes (10 points), very often (11 points), and always (13 points). A total of 49 or less points are considered “little‐to‐no impact,” between 50 and 55 “moderate impact,” between 56 and 59 “substantial impact,” and 60 or more “severe impact.” It has been shown to have a good internal consistency (Cronbach's alpha .89) and test–retest reliability (ICC ranging from 0.78 to 0.90) (Martin et al., 2004; Yang et al., 2011).

##### 2.4.1.3 Temporomandibular discomfort and pain

The ad hoc questionnaire also included four questions about the existence of TMJ discomfort (awake bruxism, sleep bruxism, chewing discomfort, and TMJ pain) with a dichotomous response (yes, no). Moreover, TMJ pain was assessed using a visual analog scale (VAS), ranging from “0” = “no pain” to “10” = “maximum pain” (Scott & Huskisson, 1979). The VAS has shown high validity and reliability for the assessment of the patient's pain intensity (confidence interval [CI] 95% = .96–.98) (Bijur et al., 2001).

#### 2.4.2 Secondary outcomes

##### 2.4.2.1 Quality of life

Measured by Cantril's Ladder of Life scale (Cantril, 1965), participants were shown an image displaying a ladder numbered from zero to ten, where the upper part of the ladder represents the best possible life for the subject (10 points), and the lower part of the ladder represents the worst possible life (= 0 points). The question which was asked was: Where on the ladder do you feel that you are at the moment? However, in our study we adapted it using two ladders. The question for one ladder was: In which step do you think you are on without using a mask? (the top step being 10 and the bottom step 0), and in the other, the question was: In which step do you think you are on using a mask? (the top step being 10 and the bottom step 0). The test–retest analyses showed acceptable agreement (Pearson's correlations between .58 and .70) in adolescents (Levin & Currie, 2014).

### 2.5 Statistical analysis

Sociodemographic variables of the participants were analyzed. The data was summarized using frequency counts, descriptive statistics, summary tables, and figures. Immediately after, Pearson's *r* for all study variables was calculated. Based on prior findings, it was expected for dependent variables to be significantly correlated. Accordingly, a repeated measures multivariate analysis of (co)variance, or RM‐MANCOVA were applied, with IBM's SPSS 27.

The advantage of using a general linear model against several linear regressions is that it allows correcting for the correlation between dependent variables. Further, if the model proves to be trustworthy, then correct means for each group can be easily derived from the model (i.e., estimated marginal means or EMM). Thus the EMM for each group were calculated and Bonferroni's correction conducting pairwise comparisons was used.

To test the hypotheses, a general linear model was specified, entering (a) existence of headache, (b) headache impact scores, and (c) QoL scores, as dependent variables, and participant's sex, frequency of headache, and daily mask use as statistical controls mask type (coded as 0 = cloth mask; 1 = surgical mask, and 2 = FFP2 mask) was entered as between‐subjects factor. Similarly, mask use as 0 = “No” and 1 = “Yes” was entered as a within‐subject factor. Finally, it was requested to SSPS to construct a between–within subject interaction term to explore if the strength of any potential effect of mask use would be contingent on the mask type employed.

The results of a RM‐MANCOVA can be interpreted as follows. First, the trustworthiness of the overall model must be determined. A nonsignificant Box's *M* test would ensure that the multivariate solution is trustworthy, as this test checks if the model violates the assumption of homogeneity of variance across groups. Second, any predictor with a significant *p*‐value for the Wilks’ lambda has a statistically significant effect on all four dependent variables in the model (headache, TMJ pain, headache intensity and QoL). Third, a significant *p*‐value of the between–within interaction term would indicate that the effect of any within‐subject factor (i.e., CMU) in the dependent variables will vary across the different between‐subject conditions (i.e., mask type).

Lastly, because an RM‐MANCOVA reports partial eta squared (partial *η* ^2^) as an indicator of effect size, any partial *η* ^2^ scores can be transformed into Cohen's *d* effect sizes to ease its interpretation. According to Cohen's method, the magnitude of the effect was classified as small (.20−.49), medium (.50−.79), or large (.80) (Cohen, 1992).

## 3 RESULTS

**FIGURE 1:**
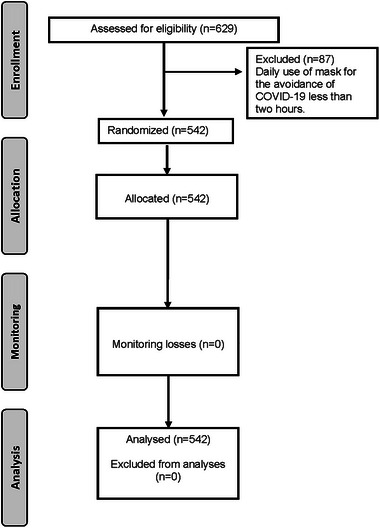
Flow chart of the study.

The final sample consisted of 542 participants (Figure 1). No attrition was identified in the sample, and thus all participants were included in the analyses. Mean age of participants was 37.23 ± 14.98 years. Regarding demographic characteristics, most participants were married (48.7%) and had university education (74.7%). Table 1 details the sociodemographic data, such as participants’ occupation at the time of the study.

**TABLE 1:** Sociodemographic data

	Mean ± SD/*n* (%) (*n* = 542)
Age (years)	37.89 ± 15.12
Sex	
Women	351 (64.7)
Men	191 (35.3)
Marital status	
Single	246 (45.3)
Married	264 (48.7)
Divorced	25 (4.6)
Widow	7 (1.2)
Education	
Primary	26 (4.8)
Secondary	111 (20.3)
University	405 (74.7)
Occupation	
Self‐employed/professional	207 (33.0)
University student	159 (25.3)
Services sector	93 (14.8)
Housework related activities	29 (4.6)
Manufacturing	28 (4.5)
Public/government employee	25 (4.0)
Retired/senior	19 (3.0)
School teacher	12 (1.9)
University professors	9 (1.4)
Health care professionals	9 (1.4)
Construction workers	8 (1.3)
Retail	6 (1.0)
Unemployed	6 (1.0)
Agricultural sector	4 (0.6)
Transportation	3 (0.5)
Clerical	2 (0.3)
First responders	2 (0.3)
Food and beverage	1 (0.2)
N/A	1 (0.2)
Face mask type	
Cloth	63 (11.6)
Surgical	328 (60.5)
FFP2	151 (27.8)

Regarding the existence of headache comparison between wearing a mask or not, 6.3% more participants reported suffering from headaches with CMU (37.3% CMU vs. 31.0% MFL). Among participants who reported headache with CMU (*n* = 202), 77.7% presented a gradual onset of headache, the most common frequency being “once a week” (63.4%). Regarding headache impact as measured by the HIT‐6, the mean score was 45.56 (SD = 8.55) when participants did not use a mask (MFL) and 47.46 (SD = 9.65) for when participants started using masks (CMU). In terms of TMJ discomfort, almost half of the participants reported awake bruxism (42.8%), but not sleep bruxism (73.4%) with CMU. Similarly, for TMJ pain (VAS scale), the mean score was 1.56 (SD = 2.27) when not using a mask and 2.28 (SD = 2.74) when participants started using masks (CMU). Finally, the mean score for QoL without using masks was 8.26 (SD = 1.90), whereas the mean score was 5.37 (SD = 2.41) when participants started using masks (CMU). Tables 2 and 3 provide more detailed frequency and descriptive statistics.

**TABLE 2:** Existence and characteristics of headache, and temporomandibular joint (TMJ) discomfort variables with continuous use of mask

Variables	*n* and %
*Headache characteristics*	
Frequency	
Once a month	49 (24.3)
One a week	128 (63.4)
Once a day	25 (12.3)
Onset	
Gradual	157 (77.7)
Sudden	45 (22.3)
Description	
Pressing/tightening quality	129 (63.9)
Pulsating quality	73 (36.1)
Intensity	
Low	80 (39.6)
Moderate	99 (49.0)
Intense	23 (11.4)
Duration	
Minutes	38 (18.8)
Hours	143 (70.8)
Days	21 (10.4)
Remission	
Progressively	162 (80.2)
Abruptly	11 (5.5)
No remission	29 (14.3)
Location	
Occipital	25 (12.4)
Frontal	108 (53.5)
Orbicular	58 (28.7)
Vertex	11 (5.4)
Time of onset	
Morning/afternoon	44 (21.8)
Evening	107 (52.9)
Night	51 (25.2)
*TMJ discomfort*	
Awake bruxism	232 (42.8)
Sleep bruxism	144 (26.6)
Chewing discomfort	39 (7.2)
TMJ pain	43 (6.8)

*Note*: The number of subjects for each variable depends on the existence and characteristics of headache and TMJ discomfort.

**TABLE 3:** Means, standard error (SE), and Pearson product‐moment correlations for all variables in this study

	Mean	SD	1.	2.	3.	4.	5.	6.	7.	8.	9.	10.	11.	12.
1. Preexisting chewing issues	.07	.25	–											
2. Self‐reported headache frequency	.37	.80	.07	–										
3. Mask average daily use (percentage)	.17	.11	.002	.09*	–									
4. Self‐reported headache—before mask use	.30	.46	.07	.30**	.02	–								
5. Self‐reported headache—during mask use	.36	.48	.09*	.93**	.11**	.37**	–							
6. VAS—TMJ—before mask use	1.56	2.27	.39**	.22**	.01	.18**	.23**	–						
7. VAS—TMJ—during mask use	2.28	2.74	.14**	.16**	.04	.08	.14**	.29**	–					
8. HIT—before mask use	45.56	8.55	.09*	.42**	.04	.53**	.43**	.26**	.20**	–				
9. HIT—during mask use	47.46	9.65	.09*	.57**	.07	.44**	.56**	.22**	.21**	.84**	–			
10. Quality of life—before mask use	8.26	1.90	−.01	.05	.13*	.01	.07	.002	.02	.02	.07	–		
11. Quality of life—during mask use	5.37	2.41	−.10*	−.16**	−.02	−.08	−.16**	−.01	−.02	−.09*	−.16**	.03	–	
12. Mask type	1.14	.61	−.03	−.03	.14**	.04	−.01	−.02	.06	−.02	−.04	.04	.06	–

*Note*: Preexisting chewing issues and self‐reported headache frequency are control variables. Mask average daily use are shown as a daily percentage ranging from “0” to “1”, where “1” = 24 h.

Abbreviations: HIT, Headache Impact Test; TMJ, temporomandibular joint; VAS, visual analog scale.

**p < .01; *p < .05.

### 3.1 Multivariate and univariate analyses

Table 3 shows means, SD, and Pearson's product‐moment correlation for all study variables. As expected, the dependent variables were correlated, with bivariate Pearson correlations (*r*) values ranging from *r* = −.16 to .56. Using RM‐MANCOVA is thus justified in this study. As a reference, Cohen established that, regardless of their sign, values of ranging from *r* = .01 to .30 are considered a small effect size, values ranging from *r* = .30 to .50 are considered moderate effect sizes and any value above *r* = .50 can be considered a large effect size (Cohen, 1992).

Box's *M* test for the multivariate solution was nonsignificant (*F*(72, 176,087.57) = 1.13, n.s.), suggesting that the multivariate solution is trustworthy. As expected, the within‐subjects factor (CMU) had a large main effect on the four dependent variables (Wilk's Λ = .88, *F*(4, 587) = 20.26 *p* < .0001, partial *η* ^2^ = .12; Cohen's *d* = .74). Similarly, a smaller, yet statistically significant effect, was found on the between‐subjects factor (Wilk's Λ = .97, *F*(8, 587) = 2.00 *p* < .05; partial *η* ^2^ = .01; Cohen's *d* = .23). Finally, a small but significant between–within subject effect was also detected (Wilk's Λ = .97, *F*(8, 1174) = 2.39 *p* < .05; partial *η* ^2^ = .02; Cohen's *d* = .25). In other words, these results suggest that mask use impacts on participants’ scores for the four dependent variables (headache, TMJ pain, headache impact, and QoL), but this effect is nuanced by mask type (cloth, surgical or FFP2).

**FIGURE 2:**
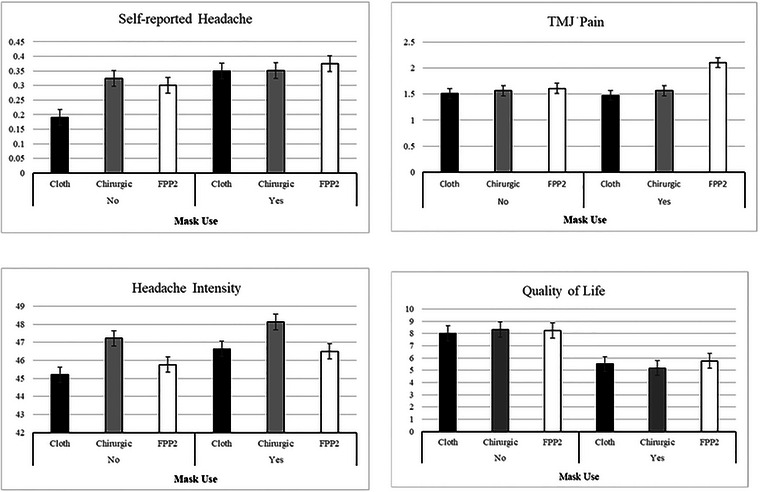
Estimated marginal means for the effect of mask use and three mask types on the studied variables.

Table 4 and Figure 2 show the EMM, SE, and 95% CI derived from the model. Headache frequency increased by 84% when participants wore cloth masks. Similarly, headache frequency increased by 25% when participants wore FFP2 masks, but there was no difference in headaches for participants who chose to wear surgical masks. The effect of mask type on the frequency of self‐reported headaches was small and in the expected direction. Alternatively, when participants wore FFP2 masks, their TMJ pain scores increased by 31%. However, that was not the case for cloth masks or surgical masks. Finally, participants’ QoL decreased substantially with mask use, regardless of the mask type. QoL decreased by 38% for surgical masks, and by 31% and 30% for cloth and FFP2 masks, respectively. These results suggest that mask type did nuance the effect of mask use on the dependent variables. To sum up, results suggest that the long‐term CMU of masks designed for short‐term use (surgical or FFP2) increases the frequency of headaches and reduces QoL.

**TABLE 4:** Estimated marginal means (EMM), standard error (SE), and 95% confidence interval (CI) for the effects of mask use and three mask types on self‐reported headache, headache intensity, temporomandibular joint (TMJ) pain, and subjective well‐being

		Condition	EMM	SE	95% CI	Mask type	EMM	SE	95% CI
Self‐reported headache	**Mask use**	No (I)	.27	.02	[.23, .32]	Cloth	.19	.05	[.10, .29]
						Surgical	.32	.02	[.28, .37]
						FPP2	.30	.04	[.23, .38]
		Yes (J)	.36	.01	[.34, .38]	Cloth	.35	.02	[.31, .39]
						Surgical	.35	.01	[.33, .37]
	*EMM difference*	I–J = −.08 (.02), p < .05*			*[−.13, −.04]*	FPP2	.38	.02	[.35, .40]
Headache impact	**Mask use**	No (I)	46.06	.38	[45.31, 46.81]	Cloth	45.20	.87	[43.49, 46.91]
						Surgical	47.22	.40	[46.43, 48.02]
						FPP2	45.76	.62	[44.53, 46.99]
		Yes (J)	47.10	.39	[46.34, 47.87]	Cloth	46.59	.89	[44.84, 48.34]
						Surgical	48.09	.41	[47.28, 48.91]
	*EMM difference*	I–J = −1.04 (.24), p < .0001*			*[−1.51, −.56]*	FPP2	46.58	.64	[45.32, 47,84]
TMJ pain	**Mask use**	No (I)				Cloth	1.51	.23	[1.05, 1.97]
			1.56	.10	[1.36, 1.76]	Surgical	1.56	.11	[1.35, 1.78]
						FPP2	1.61	.17	[1.28, 1.93]
		Yes (J)				Cloth	1.47	.30	[.88, 2.05]
			2.01	.13	[1.75, 2.26]	Surgical	2.34	.14	[2.08, 2.62]
						FPP2	2.10	.13	[1.79, 2.64]
	*EMM difference*	I–J = −.45 (.14), p < .01*							
Quality of life		No (I)				Cloth	8.01	.21	[7.59, 8.43]
	**Mask use**		8.19	.09	[8.01, 8.37]	Surgical	8.32	.10	[8.13, 8.52]
						FPP2	8.24	.15	[7.93, 8.54]
		Yes (J)				Cloth	5.50	.27	[4.97, 6.02]
			5.48	.12	[5.25, 5.71]	Surgical	5.18	.12	[4.94, 5.43]
						FPP2	5.76	.19	[5.38, 6.13]
	*EMM difference*	I–J = −2.71 (.15), p < .0001*							

*Note*: EMM refers to corrected means taking into account the correlation between dependent variables.

## 4 DISCUSSION

The main findings were that the CMU produced significant changes in headache, TMJ discomfort and pain, as well as in headache impact and QoL. In terms of mask type in CMU, it was noted that participants used surgical masks to a greater extent, followed by FFP2 and to a lesser extent, cloth masks. CMU affected to a greater or lesser extent the different variables, such as headache, headache impact, TMJ discomfort, and pain depending on the mask type, and all mask types affected QoL.

In addition, in the case of headache, onset was gradual with CMU, and the frequency mainly being at least one headache a week. Headaches were of an oppressive type resembling a tension‐type headache, although the specific type of headache was not collected. Moreover, the intensity of the headache was mild and moderate, in a similar percentage, coincident with the symptoms of tension‐type headache.

This study is in line with other authors, who assessed the impact of CMU on headache in health care professionals; however, our sample includes general population, with a greater sample than other studies (Cigiloglu et al., 2021; Köseoğlu Toksoy et al., 2021; Ramirez‐Moreno et al., 2021). It may be considered that the results obtained in this study could be taken into account in similar situations related with pandemic in the future, and thus adopt preventive measures and decrease the repercussion of CMU on QoL. With regard to the time of facemask use, previous studies have reported that headaches are most likely to appear in health care workers who wear N95‐type masks for more than 4 h and had previously been headache sufferers (Lim et al., 2006). The present study shows that these effects in the craniofacial sphere appear after CMU during at least 2 h a day.

Regarding the location of pain, this study details the concrete area of pain. In this regard, the pain location in our results was more focused on the frontal and facial region (anterior cranial region) followed by the occipital area, with mandibular disorders such as awake bruxism and sleep bruxism or chewing discomfort. In this sense, tension‐type headache pain concentrates in the upper and frontal part, as well as the occipital area, while our results showed a pain concentration in the frontal and also facial ocular region. Therefore, although we have not evaluated the results by type of headache, the pain reported by participants both in the frontal area and in the occipital area is related with tension‐type headache and with migraine, being both of them primary headaches. According to the results of the present study, headaches may be caused by the head straps which may create compression on the pericranial soft tissues (Arnold, 2018). This pressure could thus lead to an irritative effect on the underlying superficial sensory nerves (such as trigeminal or occipital nerve branches) which innervate face, head, and cervical region (Krymchantowski, 2010).

In terms of the TMJ pain, our results show significant differences on the VAS for CMU. To our best knowledge, there is no previous literature relating CMU with pain in this region. Orofacial pain can be related to TMJ continuous structures and mastication muscles (International Classification of Orofacial Pain, 2020). Accordingly, CMU may be related to facial pain, and/or ear lobe discomfort, due to tight‐fitting facemasks. Traction forces from the mask and the straps may lead to local tissue damage and produce an irritative effect on the underlying superficial sensory nerves (Ong et al., 2020). In addition, being a foreign object and of recent daily use, it has been noted that people tend to remove it from the face by pushing the jaw forward (“Mask Jaw” and TMJ Discomfort [Internet]^2023^; Pilcher, 2020). This gesture could occur in an attempt to release the mask from the face. These aspects, although being mild, could affect daily life in the short or long term. Thus, taking into account the results of the present study, this biomechanical effect in TMJ may cause pain due to CMU.

Moreover, our results showed a correlation between TMJ pain and headache with CMU. Other authors linked TMJ pain and headache (Di Paolo et al., 2017; Reik & Hale, 1981). The present study reaffirms this relationship with CMU that could be derived from the myofascial tension produced by the strap and could relate both. In this regard, fascial restrictions in one part of the body can cause undue tension in another part, due to fascial continuity (Schleip, 2003). Therefore, it could be considered that this fascial relationship (Schleip, 2003) between craniofacial structures and CMU can have an impact on the fascia due to the mild but continuous stimulus causing the permanent viscoelastic deformation of fascia (Schleip, 2003). This effect should be considered by clinicians in the initial assessments of patients and may explain the possible alterations before proceeding to apply a clinical treatment, and therefore obtain better results. Therefore, the application of myofascial release‐based techniques and similar techniques in headache (Ajimsha, 2011; Rezaeian et al., 2021) and in TMJ (Lecturer et al., 2016; Martins et al., 2016) could be taken into account by clinicians for the treatment of these CMU‐related effects. Myofascial clinicians believe that by restoring the length and health of restricted connective tissue, pressure can be mitigated on pain sensitive structures such as nerves and blood vessels (Ajimsha, 2011). In this regard, myofascial treatment on craniocervical muscles related to strap pressure in mask‐wearers could be considered in future research.

In the present study, a specific questionnaire to evaluate the impact of headache on normal daily activities was used, and significant differences for headache impact were found. Considering that CMU is compulsory in work or social activities which usually last several hours, the straps could be pressuring the craniocervical muscles, thus producing myofascial pain and peripheral sensitization which could explain the impact of headaches in daily life. Headaches may influence the ability to concentrate at work (Monzani et al., 2016), and they may also affect social activities (Eskin et al., 2013). Although CMU is needed in order to reduce the impact of COVID‐19 on everyday life, the influence of its use is affecting different aspects of people's life, and this should be taken into account by clinicians.

A novelty of the present study is that is has assessed QoL regarding mask use. The results showed that QoL is generally affected by CMU regardless of the mask type, and that it can be affected when using the mask 2 h or more. Taking into account that participants also reported an impact of headache, and that headaches have shown to affect QoL (Eskin et al., 2013) it is clear that CMU has an influence in daily life. Moreover, our results have reported not only that with CMU headache and TMJ pain have a close relationship, but also and impact on activities of daily living and QoL.

Although all masks have an impact on the variables, it affects them differently depending on the mask type. Thus, cloth masks affect the onset of headache and headache intensity to a greater extent. This may be due to the fact that non‐standardized materials transpire less than the approved types (surgical and FFP2), and because of lower quality gas exchange that could generate headaches. Other authors report that mask use involve decreased O_2_ and increased CO_2_, although this refers to surgical facemasks (Kisielinski et al., 2021). In the present study, FFP2 masks affect headache, but to a lesser extent than cloth masks. On the other hand, surgical masks and FFP2 masks affect to a greater extent TMJ pain, probably because fastening is firmer so they exert greater myofascial tension for a tighter fit.

Regarding public health implications of this study, in times of pandemic, mask use is necessary, however, it seems plausible that, having established the reasons behind the increase in headache and TMJ pain, suitable physiotherapy or medicine‐based solutions and treatments, exercises or corrective habits preventing mask use‐related side effects can be approached. The repercussions of mask use are a public health concern and treatment specialists must bear in mind these findings.

Undoubtedly, COVID‐19 has implied changes in daily life, different lifestyle has been adopted and several protection measures have been applied, among others, the regular use of EPP, provoking different symptoms. This relationship has been clearly studied in health care professionals, although it has not been studied in the general population. In this regard, Gurnani and Kaur (2021) suggested that it is a silent epidemic due to the increase of headaches. Thus, in line with Ong et al. (2021) there is an urgent need of addressing new designs and improve the existing options of EPP that, while complying with safety standards, also consider comfort and tolerance, including both health care professionals and general population.

### 4.1 Limitations and strengths

This study was carried out through an online questionnaire due to the pandemic to avoid travel and contact and to promote participants involvement for questionnaire completion. However, the process was not always completed by the subjects due to the amount of time required to answer. On the other hand, thanks to this tool it has been able to obtain a description of this problem, which is really an issue that requires attention and can explain symptoms that have increased with CMU. Future studies should consider using the Diagnostic Criteria for Temporomandibular Disorders (DC/TMD) (Schiffman et al., 2014) for TMD diagnosis and Oral Behavior Checklist (DC/TMD axis II) to evaluate bruxism more precisely. On the other hand, as strengths, it can be highlighted that this study focuses on general population and, for the first time, outcomes such as headache impact and QoL, location of pain and TMJ have been measured with valid tools. Thus, further studies could measure the same outcomes in health care professionals, in order to compare the results and therefore increase the knowledge, in professional or not professional areas, and adjust future treatments.

## 5 CONCLUSIONS

CMU increased the existence of headache, headache impact, TMJ pain, and reduced QoL. The most used type of mask was surgical, followed by FPP2 and cloth masks. The three types of masks had an impact in all the variables in a greater or lesser extent, but all types of masks decreased participants’ QoL.

## CONFLICT OF INTEREST STATEMENT

The authors declare no potential conflicts of interest with respect to the research, authorship, and/or publication of this article.

### PEER REVIEW

The peer review history for this article is available at https://publons.com/publon/10.1002/brb3.3077.

## Data Availability

Data are available upon reasonable request from the corresponding author.
